# Single Institution Retrospective Study to Determine Time to First True Progression in MGMT-Methylated Glioblastoma Patients Who Received Standard of Care

**DOI:** 10.3390/jcm15135073

**Published:** 2026-06-29

**Authors:** Isaac B. Ng, Ronak H. Jani, Abhishek Goyal, Andrew Pickles, Vikram C. Prabhu, Derek A. Wainwright, Kevin Barton, Jigisha P. Thakkar

**Affiliations:** 1 Department of Neurological Surgery, Stritch School of Medicine, Loyola University, Maywood, IL 60153, USAronak.jani@luhs.org (R.H.J.); vprabhu@lumc.edu (V.C.P.); 2 Department of Neurology, Stritch School of Medicine, Loyola University, Maywood, IL 60153, USA; 3 Department of Cancer Biology, Stritch School of Medicine, Loyola University, Maywood, IL 60153, USA; dwainwr@luc.edu; 4 Department of Hematology-Oncology, Stritch School of Medicine, Loyola University, Maywood, IL 60153, USA

**Keywords:** MGMT, glioblastoma, first progression

## Abstract

**Background**: MGMT-methylated glioblastomas respond well to temozolomide-based standard of care (Stupp protocol), demonstrate longer survival as compared to unmethylated tumors, and carry an increased risk of pseudo-progression. Establishing time to first true progression can serve as a non-invasive clinical reference point to distinguish true from pseudo-progression. **Objective**: To define the time to first true progression in patients with MGMT-methylated glioblastoma who were treated with the standard of care/Stupp protocol. **Methods**: We conducted a retrospective analysis from our institutional database of MGMT-methylated glioblastoma patients from 2018–2024. Time to first progression was measured from initial surgery to first true progression, as determined by a multidisciplinary team based on radiographic imaging review and/or pathology. **Results**: Fifteen patients met eligibility criteria. Median time to first progression of MGMT-methylated glioblastoma patients who received standard of care was twenty-one months. 40% of patients remained progression-free beyond thirty-six months after their initial surgery. **Conclusions**: Most patients with MGMT-methylated glioblastomas do not develop true progression within the first year and a half post-operatively. Therefore, MRI changes on surveillance scans should be carefully interpreted within this time frame. Expected timeline for true progression, alongside advanced radiographic imaging techniques and knowledge of treatment-specific pseudo-progression risk, may improve diagnostic accuracy.

## 1. Background

Glioblastoma, IDH-wildtype, is the most common and aggressive primary malignant brain tumor in adults, with a median OS of approximately sixteen months and a five-year survival rate of less than 7% despite intensive multimodal treatment [1]. Standard of care includes multidisciplinary treatment with maximal safe surgical resection followed by concurrent RT with temozolomide and subsequently six cycles of maintenance temozolomide (Stupp protocol) [2,3]. The WHO 2021 Classification of Central Nervous System Tumors fundamentally redefined glioblastoma: glioblastoma is now by definition a diffuse astrocytic glioma with no mutations in either IDH1 or IDH2 genes (IDH-wildtype), and IDH-mutant tumors previously classified as glioblastoma are now reclassified as astrocytoma, IDH-mutant, CNS WHO grade 4, given their biologically distinct behavior [4]. Importantly, 2021 WHO Classification also incorporated three molecular features—TERT promoter mutation, EGFR amplification, and combined chromosome 7 gain/chromosome 10 loss—as sufficient to diagnose glioblastoma, IDH-wildtype, even in the absence of necrosis or microvascular proliferation on histology [4]. The 2021 WHO classification demarcated IDH-mutant from IDH-wildtype disease—a necessity given the wide gap in survival between these subtypes, even those bearing the same histopathologic classification [5]. These changes in classification altered the clinical, radiological, molecular, and prognostic characteristics of glioblastoma and has direct implications for the interpretation of prior glioblastoma survival data, as older studies likely included IDH-mutant tumors that have a meaningfully different biology and prognosis [1].

The MGMT gene encodes a DNA repair enzyme that removes alkyl groups from the O6-position of guanine, thereby counteracting the cytotoxic DNA damage induced by alkylating chemotherapy agents such as temozolomide and carmustine. High levels of MGMT activity in cancer cells create a resistant phenotype by blunting the therapeutic effect of alkylating agents and may be an important determinant of treatment failure [6]. Downregulating MGMT expression by methylation of the MGMT promoter sequence increases temozolomide sensitivity [7] by impairing this repair mechanism, allowing O6-methylguanine lesions to trigger cytotoxicity and apoptosis. MGMT promoter methylation is an independent favorable prognostic factor in patients with newly diagnosed glioblastoma, irrespective of treatment assignment, and is associated with a significantly greater survival benefit from the addition of temozolomide to RT compared to RT alone [6]. MGMT promoter methylation is identified in approximately 40–45% of glioblastoma patients [8]. A separate analysis of patients with known MGMT status from the Stupp trial demonstrated that the combination of RT and temozolomide prolonged OS in MGMT-methylated patients (21.7 months) compared to RT alone, while no significant benefit was observed for MGMT-unmethylated patients [6]. In community settings, median OS for MGMT-methylated glioblastoma patients receiving standard first-line treatment has been reported at approximately 25.5 months, compared to 12.4 months for MGMT-unmethylated patients [9]. PFS in MGMT-methylated glioblastoma is approximately 8–12 months based on phase 3 trials [6,10,11,12,13,14]. A prospective study of MGMT-methylated glioblastoma by Brandes et al. in 2008 reported a 22-month median time to progression [15]. However, this study was conducted prior to WHO 2021 classification criteria for glioblastoma, which require IDH-wildtype status to be confirmed by NGS; accordingly, the cohort likely included IDH-mutant gliomas, given that IDH status was not systematically assessed at the time.

Natural history and behavior of MGMT-methylated glioblastoma is different as compared to MGMT-unmethylated glioblastoma. MGMT-methylated glioblastomas have an increased risk of pseudo-progression, improved survival, and higher risk of distant recurrence. They are also likely to have a prolonged time to first progression after standard treatment as compared to MGMT-unmethylated glioblastomas. To our knowledge, no studies have been conducted to determine time to first true progression in MGMT-methylated glioblastoma as defined by WHO 2021 classification criteria and treated with standard-of-care. We sought to define time to first true progression in patients with MGMT-methylated, IDH-wildtype glioblastoma—classified per WHO 2021 criteria—who were treated with the Stupp protocol.

## 2. Methods

We conducted a retrospective analysis at our institution from 2018 to 2024 of all MGMT-methylated glioblastoma meeting WHO 2021 classification criteria. This study received exemption from the local IRB. Informed consent was institutionally waived due to the retrospective nature of the study, which entails minimal risk to participating patients. We conducted a review of medical charts, imaging, and pathology reports.

We identified forty-three patients with MGMT-methylated glioblastoma who were followed at our institution from 2018–2024. Fifteen patients with MGMT-methylated glioblastoma who completed post-operative Stupp protocol were eligible for the study. IDH status was determined by NGS and, MGMT-methylation status was determined by PCR. Patients with MGMT-methylated glioblastoma were excluded from the study if IDH status was not determined by NGS, if they were unable to complete Stupp protocol due to intolerance, there was deviation from standard of care due to frailty or enrollment in a clinical trial, or if care was transferred to outside institutions.

Time to first progression was defined as the interval from initial surgery to first true progression as confirmed by pathology results upon re-resection or as determined by a multidisciplinary neuro-oncology team after review of radiographic imaging that factored specific details including RANO criteria, observation of serial scans and use of perfusion imaging (Table 1). Per RANO 2.0 criteria progressive disease is defined as ≥25% increase in the sum of the products of perpendicular diameters of the lesions, or ≥40% increase in volume, or a new measurable lesion [16].

Multifocal glioblastoma was defined as multiple synchronous enhancing lesions with FLAIR sequence demonstrating a communication between lesions via white matter tracts, gray matter, neurons, blood vessels, meninges, CSF, and ventricular system [17,18]. Single glioblastoma was defined as a single enhancing lesion with surrounding edema. GTR was defined as complete resection of enhancing tumor which was verified by post-operative MRI.

High dose RT field in glioblastoma treatment refers to the targeted peritumoral brain zone and surgical cavity receiving the maximum prescribed tumoricidal radiation. Local recurrence is common in the peritumoral brain zone within 2.0 cm of the pre-surgical initial lesion margin [19,20]. Distant recurrence was defined as recurrence outside the original resection cavity and the high dose RT field.

Data collected included patient age, single vs. multifocal disease, extent of resection, date of first surgery, date of first progression, date of next intervention, time to first progression from initial surgery and, presence or absence of distant recurrence at first progression (Table 2).

Outcomes included the proportion of MGMT-methylated glioblastomas diagnosed between 2018 and 2024, the prevalence of multifocal disease among MGMT-methylated patients, and the median time to first progression in MGMT-methylated glioblastoma treated with the standard of care.

## 3. Results

30% of glioblastomas at our institution were MGMT-methylated. From 2018 to 2024 we identified forty-three patients with MGMT-methylated glioblastoma. Fifteen patients met eligibility criteria. 26% of the MGMT-methylated glioblastomas were multifocal at presentation (Table 2).

Median time to first progression for MGMT-methylated glioblastomas that received standard of care was 21 months (range 12 to197 months) (Table 2). 40% of patients remained free of first progression for more than 36 months following initial surgery. Majority of such patients had a single focus of disease at presentation and had initial surgeries achieving GTR. Our patient with the longest time to first progression (197 months) had a single focus of hypermethylated glioblastoma at initial presentation. Of patients with PFS greater than 36 months, 67% were under 65 years of age.

33% patients had distant recurrence. All patients with early time to first recurrence (less than 13 months) after standard treatment had developed distant recurrence with stable original treated tumor site (Table 2). All patients with early time to first recurrence were older than 65 years at initial presentation.

MGMT quantification was not available for all patients. Two patients with hypermethylated glioblastomas had an improved outcome. One patient had time to first progression of more than 197 months after GTR plus Stupp protocol; and another patient had time to first progression of more than 36 months after biopsy plus Stupp protocol. Hypermethylated MGMT promoter for these two patients were determined by quantitative PCR (or pyrosequencing) assay.

## 4. Discussion

Pseudo-progression—a post-treatment phenomenon characterized by new or progressive MRI contrast-enhancement and/or edema mimicking tumor progression—typically appears within 12 weeks after the concurrent chemoradiation phase of the Stupp protocol [21,22]. Patients with MGMT-methylated glioblastomas respond favorably to standard-of-care temozolomide, exhibit a higher incidence of pseudo-progression (reported up to 90%) with a prolonged window during which pseudo-progression may occur, demonstrate improved survival and longer time to first true progression as compared to patients with MGMT-unmethylated glioblastoma [15,22].

A definitive diagnosis of progression can be made using biopsy or re-resection; however, their accuracy can be as low as 76% due to the extensive heterogeneity of glioblastoma, leading to sampling errors [23]. Non-invasive advanced imaging techniques are increasingly being utilized in clinical practice to differentiate true progression from pseudo-progression, this includes perfusion MRI sequences and MR spectroscopy. A meta-analysis of MRI techniques for evaluation of treatment response in high-grade glioma demonstrated superior performance of MR spectroscopy and perfusion MRI techniques over anatomical MRI sequences, supporting their use in clinical practice [24]. Recently, machine learning methods are being developed based on quantitative parameters from perfusion sequence and molecular signature and have shown promising results [25]. Response assessment in glioblastoma is governed by the RANO criteria. The updated RANO 2.0 criteria, published in 2023, addressed several limitations of the original framework. Since the incidence of pseudo-progression is high in the first 12 weeks after chemoradiotherapy for glioblastoma, occurring in up to 30–40% of patients; RANO 2.0 recommends mandatory confirmation of progression with a repeat MRI if there is concern for radiologic progression during this timeframe [16]. In the newly diagnosed setting, the post-RT MRI, rather than the post-surgical MRI, is now used as the baseline for comparison with subsequent scans [16]. Importantly, RANO 2.0 does not extend the mandatory confirmation window beyond 12 weeks for most settings. As a result, radiographic changes occurring after the first 12 weeks remain subject to clinical interpretation, and the distinction between true progression and delayed pseudo-progression—which is disproportionately relevant in MGMT-methylated patients—is not systematically addressed by current response criteria.

Shorter survival in MGMT-methylated glioblastoma patients could result from overtreatment due to misdiagnosis of true progression in patients who are actually experiencing pseudo-progression. Misinterpreting pseudo-progression may have profound consequences: patients may be subjected to unnecessary changes in treatment, including premature discontinuation of therapies that could potentially be beneficial, or overestimation of the efficacy of subsequent therapy. Moreover, the emotional burden on patients and their families cannot be overstated [26]. The diagnostic challenge is bidirectional: pseudo-progression misdiagnosed as true progression leads to premature cessation of potentially effective treatments and substitution for less effective second-line regimens, while true progression misdiagnosed as pseudo-progression increases waiting times and negatively influences treatment outcomes [27]. Adding to this complexity, even histopathological confirmation is imperfect: a study of 48 pathologists at 30 cancer centers demonstrated only marginal reproducibility when asked to provide a final diagnosis of active tumor, treatment effect, or unable to classify in histologic sections from glioblastoma patients with suspected early recurrence, with a kappa coefficient of 0.228 and maximum agreement on final diagnosis ranging from only 36% to 68% [28].

**Figure 1:**
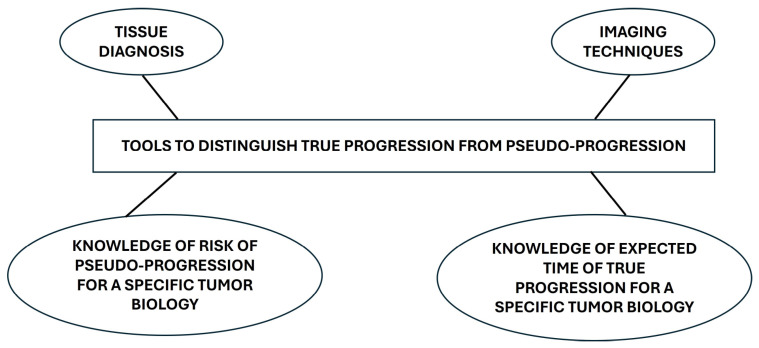
Tools to distinguish true progression from pseudo-progression. *Establishing a median time to first true progression* can serve as an additional non-invasive clinical reference point: radiographic progression occurring before the established median time to first true progression may more likely represent pseudo-progression, while progression occurring after this window raises greater concern for true progression. *Knowledge of pseudo-progression risk for a tumor biology based on treatment modality and treatment timing.* Pseudo-progression—a post-treatment phenomenon characterized by new or progressive MRI contrast-enhancement and/or edema mimicking tumor progression—typically appears within 12 weeks after the concurrent chemoradiation phase of the Stupp protocol in glioblastoma. Higher risk of pseudo-progression is seen in MGMT methylated glioblastoma. *Imaging techniques*. Perfusion-Weighted Imaging: True tumor progression exhibits high vascularization and elevated cerebral blood volume, whereas pseudo-progression typically shows minimal perfusion. Diffusion-Weighted Imaging: This method measures the movement of water molecules, determining the Apparent Diffusion Coefficient (ADC). High cellularity in true progression restricts water movement (lower ADC), while the tissue breakdown seen in pseudo-progression allows for increased water diffusion (higher ADC). Positron Emission Tomography: Use of radiotracers to measure tumor metabolism. Active, true-progressing tumors show high tracer uptake, whereas pseudo-progression (inflammatory changes) exhibits low tracer uptake perfusion. MR spectroscopy: Evaluate molecular composition of tissue by measuring metabolites like choline and N-acetylaspartate to differentiate tumors from non-cancerous lesions. *Tissue diagnosis*: A definitive diagnosis of progression can be made after biopsy or re-resection of tumor followed by histologic and molecular analysis of tissue.

Misdiagnosis of true progression in patients with pseudo-progression compromises survival as a result of overtreatment or discontinuation of an effective treatment. Therefore, it is crucial to accurately diagnose pseudo-progression in MGMT-methylated glioblastomas that are known to exhibit this phenomenon at a higher rate. There are limited non-invasive diagnostic tools to distinguish true progression from pseudo-progression (Figure 1). Establishing a median time to first true progression can serve as an additional non-invasive clinical reference point: radiographic progression occurring before the established median time to first true progression may more likely represent pseudo-progression, while progression occurring after this window raises greater concern for true progression. This contextual information, alongside advanced radiographic imaging techniques and knowledge of treatment-specific pseudo-progression risk, may improve diagnostic accuracy [29,30,31]. More precise identification of pseudo-progression could reduce misdiagnosis of true progression and decrease unnecessary early aggressive interventions—such as second-line chemotherapy, re-irradiation, or repeat surgery—in patients with MGMT-methylated glioblastomas.

Our study patients with a prolonged time to first progression (more than 36 months) following initial surgery, 86% underwent GTR and 50% received intra-operative carmustine wafer placement. Carmustine wafers are biodegradable copolymers discs impregnated with the alkylating agent (Bis-ChloroethylNitrosoUrea: BCNU). In 2003, carmustine wafer implantation in newly diagnosed glioblastoma was introduced as a therapeutic bridge during the period between tumoral surgical resection and chemoradiotherapy onset, with the aim of interfering with potential tumor growth at resection margins [32]. Several studies have demonstrated survival advantages in glioblastoma with carmustine wafer placement [33,34,35]. However, its use is a controversial topic in neurosurgery due to the lack of phase 3 studies, risk of complications (infection, pseudo-progression, CSF leak) and its use precludes clinical trial enrollment (use of carmustine wafer could give rise to confounding results). MGMT-methylated patients have a higher survival benefit with carmustine wafer placement. Studies have demonstrated long-term survival (>36 months) [36] as well as longer OS in MGMT-methylated patients who received carmustine wafer + Stupp protocol [37]. A prospective study of 111 French patients treated carmustine wafer + Stupp protocol demonstrated a longer OS in MGMT-methylated as compared to MGMT-unmethylated glioblastomas (21.7 vs. 15.1 months, *p* = 0.025) [38]. Attempting maximal safe resection with carmustine wafer in selected cases by experienced neurosurgeons may optimize clinical outcomes in MGMT-methylated glioblastomas [32,35].

Contemporary literature proposes the role of surgery beyond cytoreduction. Surgery is a biologically active event that profoundly reshapes tumor evolution, treatment response and reprograms the residual tumor microenvironment by inducing inflammation, hypoxia, vascular remodeling, immune modulation, and extracellular matrix reorganization [39]. Hypoxia-inducible signaling pathways, including HIF-1α-mediated transcriptional programs, are upregulated in residual tumor cells located near the resection margin. Hypoxic conditions are well recognized as potent drivers of tumor aggressiveness, invasion, and resistance mechanisms that potentially influence early recurrence patterns [39]. Extent of resection may influence patterns of recurrence and response to adjuvant therapies. Larger residual tumor mass may maintain pre-existing tumor–microenvironment interactions while simultaneously experiencing surgery-induced inflammatory and hypoxic stress. On the other hand, minimal residual tumor volume in peri-cavitary microenvironment may contain relatively fewer tumor cells but may still harbor infiltrative populations along white matter tracts [39]. Placement of carmustine wafer may help reduce residual tumor cells in the peri-cavitary microenvironment.

Recurrence patterns in glioblastoma is classified as local recurrence or non-local/distant recurrence based on the distance from the original resection cavity and the high dose RT field [19,20,40,41]. Patients with positive MGMT promoter methylation status have higher rate of distant recurrence which is likely due to better local control in these patients with temozolomide which is a RT sensitizer [40,42]. Temozolomide has systemic activity as well as higher response rates in MGMT-methylated patients [6]. Distant recurrence is related to various factors including presence of MGMT promoter methylation, complete resection of enhancing tumor, preoperative tumor volume (>50 cc), post-surgical ischemia and tumor involvement of the subventricular zone [6,41,43,44,45,46,47]. Post-operative ischemic volume and hypoxia might introduce an infiltrative tumor growth with diffuse and more distant tumor recurrence patterns [47,48]. All our patients with early first recurrence after standard treatment had distant recurrence which supports existing literature of better local control of MGMT-methylated glioblastoma with current standard treatment. Understanding risk factors leading to distant recurrence will help plan therapeutic strategies to prevent distant failure in MGMT-methylated glioblastomas [42].

MGMT quantification by quantitative assays methyl-BEAMing and Bs-pyrosequencing outperform methylation-specific PCR by providing better prediction of treatment response and supporting its clinical use to predict response to alkylating agents [49]. Some studies demonstrated a linear association of survival with cumulative numbers of methylated CpG sites, especially in patient who received temozolomide [50]. However, subsequent studies found that relationship between the extent of MGMT promoter methylation and survival in glioblastoma may be nonlinear and could be influenced by potential CpG hotspots and the extent of methylation at each CpG site [51,52]. Low-level promoter methylation being prognostically disadvantageous and the highest levels of promoter methylation were less beneficial than medium levels of promoter methylation [51]. Our patient with the longest time to first progression (197 months) was MGMT-hypermethylated. Further studies are warranted to clarify relationship between the extent of MGMT promoter methylation and survival in glioblastoma.

Limitations of our study include the retrospective nature of the study and the small sample size. We used strict eligibility criteria for reducing study bias. We plan extend this study to a larger patient population to improve accuracy of results. We will collaborate with other comprehensive neuro-oncology centers and include patients beyond 2024 treated at our institution.

## 5. Conclusions

Our study showed that MGMT-methylated glioblastomas exhibit a prolonged median time to progression (21 months) with standard of care. This information enhances interpretation of early surveillance imaging findings and may provide an additional non-invasive tool for differentiating pseudo-progression from true disease progression. Accordingly, surveillance imaging in patients with MGMT-methylated glioblastomas should be interpreted with particular caution during the first 21 months following standard of care therapy.

Understanding tumor biology, tumor behavior, careful interpretation of surveillance imaging, revolutions in molecular pathology, redefining roles of existing treatments and innovations in biomarker driven treatment strategies (including targeted treatment and immunotherapy) has improved survival of glioblastoma patients. Novel nanomedicine approaches and liposome-mediated targeted drug delivery holds great potential to revolutionize cancer therapy by improving drug delivery, overcoming multidrug resistance, enabling tumor-specific targeting and minimizing systemic toxicity [53,54]. Nanoparticles like hyaluronic acid nanospheres that have immunoneutral, biocompatible, biodegradable properties are able to encapsulate large drug molecules and provide specific transportation across the blood brain barrier via specific ligands attached to their surface [54]. Our study contributes to glioblastoma non-invasive diagnostics alongside with advanced radiographic imaging techniques and knowledge of treatment-specific pseudo-progression risk, to improve diagnostic accuracy.

## Figures and Tables

**Table 1:** Determination of tumor progression.

Case Number	Details of Determination of Progression
1	Surveillance MRI with new ependymal enhancement outside the high dose radiation field (left occipital horn; original tumor was left parietal). Progression of ependymal enhancement on subsequent short interval MRI scan meeting RANO criteria for tumor progression.
2	Surveillance MRI with new enhancement outside the high dose radiation field (left temporal; original tumor was right occipital), original tumor site was stable. Perfusion imaging revealed increased CBV at the new lesion.
3	Surveillance MRI with new large ring enhancing tumor outside the high dose radiation field (left temporal lobe, original tumor was in left frontal lobe), original tumor site was stable. Perfusion imaging revealed increased CBV at the new lesion. Resection of new lesion revealed recurrent glioblastoma (highly cellular glial neoplasm with nuclear pleomorphism, mitotic activity, and necrosis and increased Ki-67 labeling).
4	Surveillance MRI with increased enhancement adjacent to the resection cavity (6.3 cm) meeting RANO criteria for tumor progression. Perfusion imaging revealed no increase in CBV at the enhancing lesion. Underwent re-resection; pathology revealed recurrent tumor (moderately cellular, mitotic activity, focal microvascular proliferation, and palisading necrosis, Ki-67 labeling index with patchy increase).
5	Surveillance MRI with increased enhancement around the resection cavity meeting RANO criteria for tumor progression. Underwent re-resection; pathology revealed recurrent tumor (moderately cellular glial neoplasm with nuclear pleomorphism, mitotic activity, and foci of necrosis, Ki-67 labeling index was increased).
6	Surveillance MRI with new ring enhancing tumor outside the high dose radiation field (posterior fossa, original tumor in right parietal lobe), original tumor site was stable.
7	Surveillance MRI with increased enhancement around the resection cavity and multifocal rim enhancing lesions meeting RANO criteria for tumor progression. Perfusion imaging revealed increased CBV at some lesions.
8	Surveillance MRI with increased nodular enhancement around the resection cavity and new enhancing lesions adjacent to the resection cavity meeting RANO criteria for tumor progression. Perfusion imaging revealed mildly increased CBV at enhancing lesions.
9	Surveillance MRI with increased enhancement around the resection cavity meeting RANO criteria for tumor progression. Underwent re-resection; pathology revealed recurrent glioblastoma (densely cellular pleomorphic glioma with mitotic activity, focal microvascular proliferation, and necrosis, increased Ki-67 at 25–30%).
10	Surveillance MRI with new enhancement outside the high dose radiation field (new right periventricular enhancement, original tumor was in right frontal lobe), original tumor site was stable. Perfusion imaging indeterminate as 5 mm lesion size was too small to be characterized by perfusion. Recurrence was determined after a muti-disciplinary tumor board discussion based on timing of enhancement (40 months after initial surgery) and outside high dose radiation field.
11	No recurrence *
12	No recurrence *
13	No recurrence *
14	No recurrence *
15	Surveillance MRI with increased enhancement around the resection cavity meeting RANO criteria for tumor progression. Underwent re-resection; pathology revealed recurrent glioblastoma (markedly cellular infiltrating glioma with prominent nuclear pleomorphism, increased mitoses, microvascular proliferation, and geographic and pseudo-palisading necrosis, and increased Ki67 at 30%).

Abbreviations: CBV: cerebral blood volume; MRI: magnetic resonance imaging; RANO: Response Assessment in Neuro-Oncology. All cases were discussed at multidisciplinary tumor board to determine progression. “*”: these patients did not have recurrence of tumor up to the publication of this manuscript.

**Table 2:** Median time to first progression in MGMT-methylated glioblastoma.

Case Number	Age/Gender	Number of Foci	Extent of Resection	Time to First Progression (Months)	Distant Recurrence at First Progression
1	74/F	Multifocal	Biopsy	12	Yes
2	65/M	Multifocal	Biopsy	12.5	Yes
3	70/F	Single	GTR	13	Yes
4	65/M	Single	GTR + CW	14	No
5	56/M	Single	STR	15	No
6	57/M	Single	GTR	16	Yes
7	72/M	Multifocal	GTR of one lesion	21	No
8	69/F	Multifocal	GTR of one lesion	21	No
9	59/M	Single	GTR	27	No
10	68/F	Single	GTR + CW	40	Yes
11	65/F	Single	GTR	* 36+	No
12	44/F	Single	GTR + CW	* 36+	No
13	58/M	Single	Biopsy	* 36+	No
14	60/F	Single	GTR + CW	* 36+	No
15	41/F	Single	GTR	197	No

Abbreviations: GTR, gross total resection; CW, carmustine wafers; STR, subtotal resection. * Patients that have not has first progression up to this manuscript publication.

## Data Availability

The datasets generated during and/or analyzed during the current study are available from the corresponding author on reasonable request.

## Abbreviations

MGMT	O6-methylguanine-DNA methyltransferase
MRI	Magnetic Resonance Imaging
FLAIR	Fluid-Attenuated Inversion Recovery
RT	Radiation Therapy
OS	Overall Survival
PFS	Progression Free Survival
IDH	Isocitrate Dehydrogenase
DNA	Deoxyribonucleic Acid
TERT	Telomerase Reverse Transcriptase
EGFR	Epidermal Growth Factor Receptor
IRB	Institutional Review Board
NGS	Next Generation Sequencing
PCR	Polymerase chain reaction
RANO	Response Assessment in Neuro-Oncology
WHO	World Health Organization
GTR	Gross Total Resection
HIF	Hypoxia-Inducible Factors
CSF	Cerebrospinal Fluid
